# Evaluation of the INTERGROWTH-21^st^ project newborn standard for use in Canada

**DOI:** 10.1371/journal.pone.0172910

**Published:** 2017-03-03

**Authors:** Shiliang Liu, Amy Metcalfe, Juan Andrés León, Reg Sauve, Michael S. Kramer, K. S. Joseph, for the Canadian Perinatal Surveillance System (Public Health Agency of Canada)

**Affiliations:** 1 Maternal, Child and Youth Health, Surveillance and Epidemiology Division, Centre for Chronic Disease Prevention, Public Health Agency of Canada, Ottawa, Canada; 2 Department of Obstetrics and Gynecology, University of Calgary, Calgary, Canada; 3 Department of Community Health Sciences, University of Calgary, Calgary, Canada; 4 Department of Pediatrics, University of Calgary, Calgary, Canada; 5 Departments of Pediatrics and of Epidemiology, Biostatistics and Occupational Health, McGill University, Montreal, Canada; 6 Department of Obstetrics and Gynaecology and the School of Population and Public Health, University of British Columbia and the Children’s and Women’s Hospital and Health Centre of British Columbia, Vancouver, Canada; Centre Hospitalier Universitaire Vaudois, FRANCE

## Abstract

**Objective:**

To evaluate the performance of the INTERGROWTH-21^st^ Project newborn standard vis-a-vis the current Canadian birth weight-for-gestational age reference.

**Methods:**

All hospital-based singleton live births in Canada (excluding Quebec) between 2002 and 2012 with a gestational age between 33 and 42 weeks were included using information obtained from the Canadian Institute for Health Information. Small- and large-for gestational age centile categories of the INTERGROWTH standard and Canadian reference were contrasted in terms of frequency distributions and rates of composite neonatal morbidity/mortality.

**Results:**

Among 2,753,817 singleton live births, 0.87% and 9.63% were <3^rd^ centile and >97^th^ centile, respectively, of the INTERGROWTH standard, while 2.27% and 3.55% were <3^rd^ centile and >97^th^ centile, respectively, of the Canadian reference. Infants <3^rd^ centile and >97^th^ centile had a composite neonatal morbidity/mortality rate of 46.4 and 12.9 per 1,000 live births, respectively, under the INTERGROWTH standard and 30.9 and 16.6 per 1,000 live births, respectively, under the Canadian reference. The INTERGROWTH standard <3^rd^ centile and >97^th^ centile categories had detection rates of 3.14% and 9.74%, respectively, for composite neonatal morbidity/ mortality compared with 5.48% and 4.60%, respectively for the Canadian reference. Similar patterns were evident in high- and low-risk subpopulations.

**Conclusions:**

The centile distribution of the INTERGROWTH newborn standard is left shifted compared with the Canadian reference, and this shift alters the frequencies and neonatal morbidity/mortality rates associated with specific centile categories. Further outcome-based research is required for defining abnormal growth categories before the INTERGROWTH newborn standard can be used.

## Introduction

The INTERGROWTH-21^st^ Project recently published international standards for fetal growth and newborn size created using a rigorous methodology [1,2]. These fetal growth and newborn size standards complement the World Health Organization’s Child Growth Standards [3] and permit growth monitoring from early gestation through childhood. A key aspect of the INTERGROWTH-21^st^ Project was the selection of a healthy cohort of fetuses from normal pregnancies in order to ensure that the resultant standard provides normative and prescriptive centiles of fetal and newborn growth. INTERGROWTH-21^st^ project investigators suggest that their fetal and newborn growth standards will serve as worldwide standards for fetuses and newborns [1,2,4].

The INTERGROWTH-21^st^ Project newborn birth weight-for-gestational age standard is based on the results of the multicentre, multiethnic, Newborn Cross-Sectional Study in which 20,486 eligible pregnant women were recruited from 8 sites worldwide [1]. Inclusion criteria ensured a healthy study population including (among others) site parameters related to altitude and pollution, and individual level factors such as maternal age, height, body mass index, smoking status, and medical and obstetric history. All women had ultrasound confirmed gestational age, and anthropometric measurements on the infants were completed within 12 hours after birth. The measurement devices used for anthropometry were calibrated twice weekly, and all measurements were standardized. Smoothed centiles of birth weight, length and head circumference were estimated at each gestational week between 33 and 42 weeks gestation using robust statistical techniques [2].

The rigorous design and analysis methods of the INTERGROWTH-21^st^ Project notwithstanding, the clinical and public health consequences of switching from currently used fetal growth and newborn references to the INTERGROWTH-21^st^ standards are unclear. We carried out an evaluation of the performance of the INTERGROWTH-21^st^ Project birth weight-for-gestational age standard vis-a-vis the current Canadian birth weight-for-gestational age reference [5] by contrasting the frequency of centile categories for identifying growth abnormalities (including both small- and large-for-gestational age live births), and the rates of severe neonatal morbidity and neonatal mortality within these centile categories.

## Methods

We carried out a retrospective cohort study of all singleton live births in Canada (excluding Quebec) for the period April 2002 to March 2013 (fiscal years 2002 to 2012) using information obtained from the Discharge Abstract Database of the Canadian Institute for Health Information. This database contains records of all hospitalizations in Canada (excluding Quebec) and includes approximately 98% of all births [6]. The collated data were abstracted from medical charts by trained medical records personnel using standardized definitions and included information on gestational age (obstetric estimate), birth weight, and maternal and newborn diagnoses and interventions during the childbirth admission. During the study period all diagnoses were coded using the International Classification of Diseases 10th revision, Canadian modification (ICD-10 CA), while procedures were coded using the Canadian Classification of Health Interventions (CCI). Information in the database is routinely checked for accuracy, and validation studies have shown the information to be complete and accurate [7].

Live births between 33 and 42 weeks gestation were categorized into the conventional birth weight-for-gestational age centile categories for identifying small-for-gestational age live births, appropriate-for-gestational live births and large-for-gestational age live births using both the INTERGROWTH newborn standard [1] and the Canadian reference [5] (hereafter referred to as the INTERGROWTH and Canadian criteria). Centile categories of interest included birth weight for gestational age <3^rd^ centile, 3^rd^ to <10^th^ centile, 10 to <50^th^ centile, 50^th^ to 90^th^ centile, >90^th^ to 97^th^ centile and >97^th^ centile. Frequency distributions of live births in the early and later years of the study period (2002–03 and 2011–12) and over the entire study period (2002–2012), were compared using centile categories based on both the INTERGROWTH and Canadian criteria. The frequency distribution of live births in specific low- and high-risk subpopulations were also compared within the respective centile categories. The high-risk categories of interest included infants affected by maternal hypertensive disorders (P00.0), infants of mothers with gestational diabetes (P70.0), and infants of diabetic mothers (P70.1). The low-risk categories included all live births and live births not affected by slow growth/malnutrition (P05), maternal hypertension (P00.0) or diabetes (P70.0).

Rates of severe neonatal morbidity (i.e., neonatal seizures (P90), assisted ventilation (1GZ31CAND, and 1GZ31CRND), assisted ventilation including continuous positive airway pressure (1GZ31CBND) and birth asphyxia (P20.1, P20.9 and P21)), and neonatal mortality were calculated among live births in the different centile categories based on the INTERGROWTH [1] and Canadian criteria [5]. Rates of composite neonatal morbidity (seizures, assisted ventilation, continuous positive airway pressure or birth asphyxia) and neonatal mortality were also estimated. The prognostic performance of the INTERGROWTH and Canadian criteria were also assessed in terms of the ability of the small- and large-for-gestational age centile categories to identify composite severe neonatal morbidity/mortality using rate ratios with 95% confidence intervals (CI) and detection (sensitivity) rates [8].

The study was carried out by the Canadian Perinatal Surveillance System of the Public Health Agency of Canada under its health surveillance mandate using publicly accessible anonymized data. Statistical analyses were carried out using SAS version 9 (Cary, NC).

## Results

The study included 2,753,817 singleton live births. Overall between 2002 and 2012, the birth weight-for-gestational age of 0.87% of infants was <3^rd^ centile of the INTERGROWTH criteria, compared with 2.27% of infants who were <3^rd^ centile under the Canadian criteria (P<0.001, Table 1). Similarly, a substantially lower proportion of live births were <10^th^ centile of birth weight-for-gestational age of the INTERGROWTH criteria compared with the Canadian criteria. In contrast, a substantially larger proportion of live births were >97^th^ and >90 centile under the INTERGROWTH criteria compared with the Canadian criteria (9.63% and 3.55% were >97^th^, respectively, P<0.001). The patterns were evident both in 2002–03 and 2011–12 (Table 1).

**Table 1:** Numbers and proportions of live births in birth weight-for-gestational centile categories of the INTERGROWTH -21^st^ Project standard and the Canadian reference, Canada, 2002–12.

Period/	Live	<3^rd^		3^rd^-<10^th^		10^th^-<50^th^			50^th^-90^th^		>90^th^-97^th^		>97^th^	
Standard or reference	Births	centile		centile		centile			centile		centile		centile	
		No.	(%)	No.	(%)	No.	(%)		No.	(%)	No.	(%)	No.	(%)
All live births 2002–12														
INTERGROWTH std.	2,753,817	23,885	0.87	71,237	2.59	673,319	24.5		1,329,699	48.3	390,471	14.2	265,206	9.63
Canadian ref.	2,753,815	62,558	2.27	171,810	6.24	1,067,272	38.8		1,142,574	41.5	211,734	7.69	97,867	3.55
All live births 2002–03														
INTERGROWTH std.	398,327	3,688	0.82	10,399	2.32	95,176	21.3		190,713	42.6	57,823	12.9	40,528	9.05
Canadian ref.	398,327	9,496	2.12	24,412	5.45	151,233	33.8		166,108	37.1	31,999	7.15	15,079	3.37
All live births 2011–12														
INTERGROWTH std.	546,897	4,527	0.83	14,776	2.70	137,918	25.2		265,188	48.5	74,981	13.7	49,507	9.05
Canadian ref.	546,897	12,582	2.30	35,555	6.50	216,866	39.7		223,769	40.9	40,071	7.33	18,054	3.30
Infants affected by maternal hypertensive disorders 2002–12														
INTERGROWTH std.	6,217	351	5.65	568	9.14	1,919	30.9		2,188	35.2	638	10.3	553	8.89
Canadian ref.	6,217	579	9.31	867	14.0	2,245	36.1		1,879	30.2	370	5.95	277	4.46
Infants of mothers with gestational diabetes 2002–12														
INTERGROWTH std.	24,788	324	1.31	715	2.88	4,769	19.2		9,147	36.9	3,473	14.0	6,360	25.7
Canadian ref.	24,788	686	2.77	1,434	5.79	7,034	28.4		8,640	34.9	2,945	11.9	4,049	16.3
Infants of mothers with pre-existing diabetes 2002–12														
INTERGROWTH std.	10,521	99	0.94	199	1.89	1,266	12.0		2,945	28.0	1,605	15.3	4,407	41.9
Canadian ref.	10,521	178	1.69	409	3.89	1,922	18.3		3,230	30.7	1,668	15.9	3,114	29.6
Infants without slow growth/malnutrition or maternal hypertension/diabetes 2002–12														
INTERGROWTH std.	2,659,785	10,627	0.39	51,312	1.86	644,779	23.4		1,314,372	47.7	384,735	14.0	253,960	9.22
Canadian ref.	2,659,785	38,554	1.40	147,720	5.36	1,047,779	38.8		1,128,473	41.5	206,751	7.51	90,506	3.29

Among infants affected by maternal hypertensive disorders, the proportion of live births <3^rd^ centile was 5.65% under the INTERGROWTH criteria, while the same proportions were 9.31% under the Canadian criteria. Of the infants of mothers with gestational diabetes 1.31% were classified as <3^rd^ centile by the INTERGROWTH criteria, while 2.77% were similarly classified by the Canadian criteria. On the other hand, 25.7% of the infants of mothers with gestational diabetes were >97^th^ centile under the INTERGROWTH criteria, while 16.3% were classified as >97^th^ under Canadian criteria. Infants of mothers with pre-existing diabetes showed similar patterns (Table 1) and essentially similar patterns were observed in the 3^rd^ to <10^th^ centile and the >90^th^ to 97^th^ centile categories.

**Fig 1:**
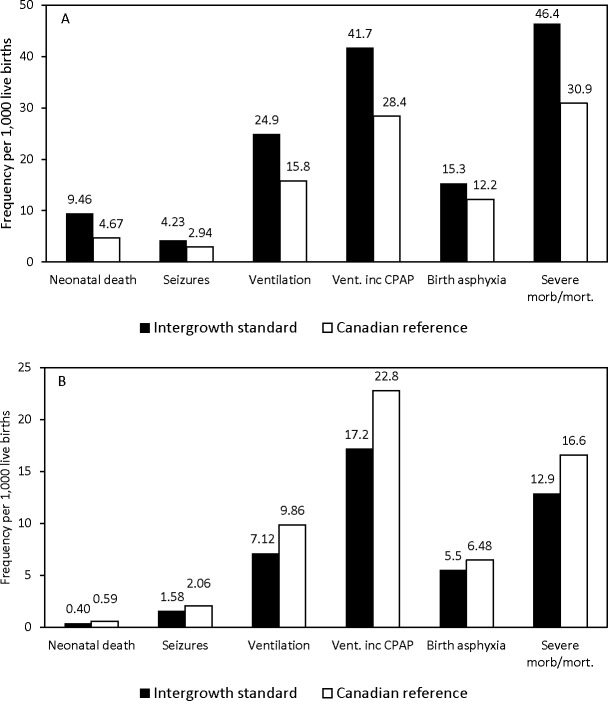
**Rates of severe neonatal morbidity and neonatal death among live births <3^rd^ centile (Panel A) and >97^th^ centile (Panel B) of the INTERGROWTH-21^st^ Project newborn birth weight-for-gestational age standard and the Canadian reference, Canada (excluding Quebec) 2002–2012.** Vent. inc CPAP denotes assisted ventilation including continuous positive airway pressure and severe morb/mort. denotes composite severe neonatal morbidity and neonatal mortality.

Table 2 shows rates of severe neonatal morbidity and neonatal death among live births within specific centile categories of the INTERGROWTH and Canadian criteria. The rate of neonatal death was two-fold higher among live births <3^rd^ centile of the INTERGROWTH criteria compared with the same rate among live births <3^rd^ centile of the Canadian criteria (9.46 vs 4.67 per 1,000 live births, P <0.001, Fig 1). The rate of neonatal death was also higher among live births between the 3^rd^ centile and <10^th^ centile of the INTERGROWTH criteria compared with the Canadian criteria (P <0.001) and among live births between the 10^th^ to the <50^th^ centile (P <0.001). However, rates of neonatal death among live births between >90^th^ centile and the 97^th^ centile of the INTERGROWTH and Canadian criteria were similar (P = 0.46), while neonatal death rates were significantly lower among live births >97^th^ centile of the INTERGROWTH criteria as compared with the Canadian criteria (P = 0.02, Fig 1). Rates of specific severe neonatal morbidity and composite neonatal mortality/morbidity followed a similar pattern (Table 2 and Fig 1).

**Table 2:** Neonatal mortality and morbidity rates (per 1,000 live births) among birth weight-for-gestational age centile categories of the INTERGROWTH-21^st^ Project standard and the Canadian reference, Canada, 2002–12.

Period/	All live births		<3^rd^		3^rd^-<10^th^		10^th^-<50^th^		50^th^-90^th^		>90^th^-97^th^		>97^th^	
Standard or			centile		centile		centile		centile		centile		centile	
reference	No.	Rate	No.	Rate	No.	Rate	No.	Rate	No.	Rate	No.	Rate	No.	Rate
INTERGROWTH standard														
Neonatal death	1,423	0.52	226	9.46	140	1.97	392	0.58	447	0.34	112	0.29	106	0.40
Seizures	3,618	1.31	101	4.23	147	2.06	968	1.44	1,524	1.15	460	1.18	418	1.58
Ventilation	18,236	6.99	595	24.9	867	12.2	5,162	7.67	8,511	6.40	2,212	5.66	1,889	7.12
Vent. inc CPAP	42,854	15.6	995	41.7	1,719	24.1	10,943	16.3	19,332	14.5	5,307	13.6	4,558	17.2
Birth asphyxia	15,196	5.52	366	15.3	717	10.1	4,269	6.34	6,535	4.91	1,851	4.74	1,458	5.50
Severe morb/mort.	35,249	12.8	1,108	46.4	1,642	23.0	9,577	14.2	15,313	11.5	4,176	10.7	3,433	12.9
Canadian reference														
Neonatal death	1423	0.52	292	4.67	186	1.08	461	0.43	357	0.31	68	0.32	58	0.59
Seizures	3,618	1.31	184	2.94	284	1.65	1,333	1.25	1,342	1.17	273	1.29	202	2.06
Ventilation	19,236	6.99	987	15.8	1,630	9.49	7,168	6.72	7,093	6.21	1,393	6.58	965	9.86
Vent. incl. CPAP	42,854	15.6	1,775	28.4	3,320	19.3	15,728	14.7	16,438	14.4	3,362	15.9	2,230	22.8
Birth asphyxia	15,196	5.52	762	12.2	1,371	7.98	5,958	5.58	5,407	4.73	1,064	5.03	634	6.48
Severe morb/mort.	35,249	12.8	1,933	30.9	3,048	17.7	13,348	12.5	12,766	11.2	2,530	12.0	1,623	16.6

Vent. incl. CPAP denotes assisted ventilation including continuous positive airway pressure, while severe morb/mort denotes composite severe neonatal morbidity or neonatal mortality. Composite severe neonatal morbidity/mortality included neonatal seizures, assisted ventilation, birth asphyxia, and neonatal mortality.

Table 3 shows the frequency and composite neonatal morbidity/mortality rates in the different centile categories among live births at 33–36 weeks and 37–42 weeks gestation. The left-shift in the frequency of live births when centiles were based on INTERGROWTH criteria compared with the Canadian criteria was observed in both categories of gestational age. Among live births at 33–36 weeks, rates of composite neonatal morbidity/mortality were non-significantly higher in all centile categories under INTERGROWTH criteria except for the large-for-gestational centile categories; composite neonatal mortality/morbidity rates were non-significantly lower in the 90^th^ to 97th centile category and significantly lower in the >97^th^ centile category under the INTERGROWTH criteria compared with the Canadian criteria (P<0.001, Table 3). Patterns of composite neonatal morbidity/mortality among live births 37–42 weeks gestation were similar to overall patterns (Table 2), with higher rates of morbidity/mortality in the small-for-gestational age centile categories under INTERGROWTH criteria and higher rates of morbidity/mortality in the large-for-gestational age centile categories under Canadian criteria. Morbidity/mortality rate differences between centile categories (e.g., <3^rd^ centile vs 50-90^th^ centile) were larger at 37–42 weeks than at 33–36 weeks under both INTERGROWTH and Canadian criteria (Table 3).

**Table 3:** Rates of composite severe neonatal morbidity/mortality and prognostic performance of the INTERGROWTH-21^st^ Project standard and the Canadian reference among all live births and among low and high risk infants, Canada, 2002–12.

Gestational age	INTERGROWTH standard				Canadian reference				INTERGROWTH vs.	
category and									Canadian criteria	
centile group	Frequency		Composite		Frequency		Composite		Rate ratio	P value
			morbidity and				morbidity and		(95% CI)	
			mortality/1000				mortality/1000			
	No.	Rate	No.	Rate	No.	Rate	No.	Rate		
**33–36 weeks**	146,669	100.0	8,056	54.9	146,669	100.0	8,056	54.9	-	-
<3^rd^	2,816	1.92	263	93.4	3,759	2.56	348	92.6	1.01 (0.87–1.18	0.91
3^rd^ to <10^th^	5,629	3.84	408	72.5	10,191	6.95	698	68.5	1.06 (0.94–1.19	0.35
10^th^ to <50^th^	40,602	27.7	2,207	54.4	57,393	39.1	3,044	53.0	1.02 (0.97–1.19)	0.37
50^th^ to 90^th^	72,225	49.2	3,824	52.9	59,100	40.3	3,011	50.9	1.04 (0.99–1.09)	0.10
>90^th^ to<97^th^	14,894	10.2	745	50.0	10,581	7.21	562	53.1	0.94 (0.85–1.05)	0.27
>97^th^	10,503	7.16	609	58.0	5,643	3.85	392	69.5	0.83 (0.74–0.94)	0.004
**37–43 weeks**	2,607,148	100.0	27,193	10.4	2,607,148	100.0	27,193	10.4		
<3^rd^	21,069	0.81	845	40.1	58,799	2.26	1,585	27.0	1.49 (1.37–1.61)	<0.0001
3^rd^ to <10^th^	65,608	2.52	1,234	18.8	161,619	6.20	2,350	14.5	1.29 (1.21–1.38)	<0.0001
10^th^ to <50^th^	632,717	24.3	7,370	11.6	1,009,879	38.7	10,304	10.2	1.14 (1.11–1.18)	<0.0001
50^th^ to 90^th^	1,257,474	48.2	11,489	9.14	1,083,474	41.6	9,755	9.00	1.01 (0.99–1.04)	0.28
>90^th^ to<97^th^	375,577	14.4	3,431	9.14	201,153	7.72	1,968	9.78	0.93 (0.88–0.99)	0.01
>97^th^	254,703	9.77	2,824	11.1	92,224	3.54	1,231	13.3	0.83 (0.78–0.89)	<0.0001

Composite neonatal morbidity/mortality included neonatal seizures, assisted ventilation, birth asphyxia, and neonatal mortality.

Table 4 highlights differences in severe neonatal morbidity/mortality within centile categories of the INTERGROWTH and Canadian criteria. Among all live births, infants <3^rd^ centile of the INTERGROWTH criteria had a rate of composite neonatal morbidity/mortality that was 4.03 times higher (95% CI 3.80–4.28) than the same rate among live births in the 50^th^ to 90^th^ centile of the same criteria. In contrast, there was a 2.77 (95% CI 2.64–2.90) times higher rate of composite severe neonatal morbidity/mortality among live births <3^rd^ centile compared with live births in the 50^th^ to 90^th^ centiles of the Canadian criteria. This difference in rate ratios was reversed in contrasts between rates of composite neonatal morbidity/mortality among large-for-gestational age infants vs infants in the 50^th^ to 90^th^ centile category; the rate ratio among all live births contrasting composite neonatal morbidity/mortality rates among those >97^th^ centile vs those in the 50^th^-90^th^ centile of the INTERGROWTH criteria was 1.12 (95% CI 1.08–1.17), while the same rate ratio with centiles based on the Canadian criteria was 1.48 (95% CI 1.41–1.56). Table 4 also shows similar patterns in contrasts between the INTERGROWTH and Canadian criteria for high and low risk infants.

**Table 4:** Rates of composite severe neonatal morbidity/mortality and prognostic performance of the INTERGROWTH-21^st^ Project standard and the Canadian reference among all live births and among low and high risk infants, Canada, 2002–12.

Infant group and	INTERGROWTH standard				Canadian reference			
category	Morbidity/	Rate	95% CI	Detection	Morbidty/	Rate	95% CI	Detection
	mortality rate	ratio*		rate (%)†	mortality rate	ratio*		rate (%)†
All live births								
<3^rd^ centile	46.4	4.03	3.80–4.28	3.14	30.9	2.77	2.64–2.90	5.48
3^rd^-<10^th^ centile	23.0	2.00	1.90–2.10	4.66	17.7	1.59	1.53–1.65	8.65
10^th^-<50^th^ centile	14.2	1.24	1.20–1.27	27.2	12.5	1.12	1.09–1.15	37.9
50^th^-90^th^ centile	11.5	1.00	-	43.4	11.2	1.00	-	36.2
>90^th^-97^th^ centile	10.7	0.93	0.90–0.96	11.8	11.9	1.07	1.03–1.12	7.18
>97^th^ centile	12.9	1.12	1.08–1.17	9.74	16.6	1.48	1.41–1.56	4.60
Infants affected by maternal hypertensive disorders								
<3^rd^ centile	74.1	6.59	4.55–9.55	6.19	79.4	7.29	5.52–9.63	11.0
3^rd^-<10^th^ centile	79.2	7.05	5.33–9.34	10.7	77.3	7.09	5.63–8.93	16.0
Infants of mothers with gestational diabetes								
>90^th^-97^th^ centile	22.6	2.33	1.90–2.86	13.0	34.6	3.18	2.63–3.85	14.6
>97^th^ centile	37.7	3.36	2.96–3.81	34.2	40.3	3.70	3.18–4.30	23.3
Infants of diabetic mothers								
>90^th^-97^th^ centile	34.9	3.11	2.40–4.02	11.6	37.8	3.47	2.72–4.42	13.0
>97^th^ centile	50.6	4.50	3.96–5.12	46.1	55.9	5.13	4.43–5.93	36.0
Infants without slow growth/malnutrition or maternal hypertension/diabetes								
<3^rd^ centile	39.7	3.53	3.21–3.89	1.33	23.6	2.16	2.02–2.31	2.86
3^rd^-<10^th^ centile	19.1	1.70	1.59–1.81	3.08	15.5	1.43	1.36–1.49	7.22
10^th^-<50^th^ centile	13.5	1.20	1.17–1.23	27.3	12.1	1.11	1.08–1.14	39.8
50^th^-90^th^ centile	11.2	1.00	-	46.4	10.9	1.00	-	38.7
>90^th^-97^th^ centile	10.4	0.93	0.89–0.96	12.6	11.4	1.04	1.00–1.09	7.40
>97^th^ centile	11.6	1.03	0.99–1.07	9.27	14.1	1.29	1.22–1.37	4.01

* Infants in the 50^th^ to 90^th^ centile category served as the reference group for all live births, while infants without slow growth/ malnutrition or maternal hypertension/diabetes who were in the 50^th^ to 90^th^ centile category served as the reference group for all other rate ratio calculations.

† Detection (sensitivity) rate refers to the proportion of composite neonatal morbidity/morbidity that occurred in that centile category.

The detection (sensitivity) rates of the INTERGROWTH criteria by centile category showed that 3.14% and 7.80% of all cases of composite neonatal morbidity/mortality occurred among live births <3^rd^ centile and <10^th^ centile, respectively, while 5.48% and 14.1% of composite neonatal morbidity/mortality occurred among live births <3^rd^ centile and <10^th^ centile of the Canadian criteria (Table 4). However, the detection rates of the >97^th^ centile category (9.74%) and the >90^th^ centile category (21.5%) under the INTERGROWTH criteria were higher than the detection rates of the >97^th^ centile category (4.60%) and the >90^th^ centile category (11.8%) under the Canadian criteria. Similar patterns were evident in comparisons of detection rates by centile categories in low- and high-risk populations (Table 4) and among infants in the 33–36 week and 37–42 week categories (Table 3).

## Discussion

Our study shows that the normative centile distribution of the INTERGROWTH birth weight-for-gestational age standard [1] is left-shifted compared with the Canadian reference [5]. The proportion of Canadian live births deemed to be small-for-gestational age by INTERGROWTH criteria is lower than the proportion identified as small-for-gestational age by the Canadian criteria, while the proportion of live births classified as large-for-gestational age is substantially higher under INTERGROWTH criteria. Differences in neonatal morbidity and mortality rates within centile categories of the INTERGROWTH and Canadian criteria are a product of the left shift in birth weight-for-gestational age centiles of the INTERGROWTH standard. Since small-for-gestational age live births identified by the INTERGROWTH criteria represent more severely growth-restricted infants, their neonatal morbidity/mortality profile is worse than that of small-for-gestational age infants identified by the Canadian criteria. Conversely, the relatively less stringent INTERGROWTH criteria for identifying large-for-gestational age infants resulted in relatively low neonatal morbidity/mortality rates among such infants. Under the normative premise of the INTERGROWTH standard, this overall picture suggests that Canadian live births have low rates of growth restriction and high rates of excess growth. Given the similarity of the Canadian birth weight-for-gestational age reference to the references of other high income countries, it is probable that these findings apply to most industrialized countries.

A large study size and a validated data source are important strengths of our study. Limitations include potential errors with regard to gestational age which would have affected birth weight-for-gestational age centiles under both the INTERGROWTH and Canadian criteria. Although most women in Canada have ultrasound-confirmed gestational age, some errors are inevitable in chart abstraction and transcription. The results of analyses among 33–36 week infants were influenced by the choice of neonatal morbidity (diseases of late gestation which are more influenced by growth restriction [9]), and also by the relatively smaller numbers of such infants. Some transcription errors are also possible with regard to the diagnoses of severe neonatal morbidity. On the other hand, previous studies [7] have shown that the database information is valid, and all diagnoses and procedures were coded using the same system (ICD-10CA and CCI) during the study period. Other study limitations include an evaluation restricted to birth weight-for-gestational age, whereas the INTERGROWTH standard (but not the Canadian reference) included other anthropometric measurements.

Significant changes in the frequency of small-for-gestational age (<3^rd^ and <10^th^ centile) and large-for-gestational age (>97^th^ and >90^th^ centile) live births would occur if the INTERGROWTH standard replaced the Canadian reference (assuming the centile cutoffs for identifying these high risk subpopulations remained unchanged). Another important finding of our study is the decline in rates of severe neonatal morbidity and neonatal death with increasing birth weight-for-gestational age from the <3^rd^ centile category to the >90-97^th^ centile category. In fact, this decline in perinatal morbidity/mortality with increasing birth weight-for-gestational age centile has been demonstrated in numerous previous studies [10–17] and supports the case for an outcome-based determination of INTERGROWTH centile cut-offs for surveillance and monitoring of abnormal growth [18]. The identification of optimal cut-offs for identifying infants at high risk for adverse outcomes needs to balance the proportion of severe neonatal morbidity and neonatal death identified by the cut-off (sensitivity) with the proportion of infants deemed to be high risk (stratification capacity [8]). The optimal INTERGROWTH standard centile cut-offs for identifying small- and large-for-gestational age live births will likely be resource/cost dependent and hence spatio-temporally specific.

The INTERGROWTH-21^st^ Project suggests that normative standards based on healthy fetuses and newborns are universal and do not need to be customized by ethnicity [4]. Although this conclusion has been criticised [19–22], the INTERGROWTH-21^st^ Project findings are compelling because of the meticulous design and rigorous analysis and also because observed differences in between-country and ethnic-specific fetal growth references potentially reflect differences in maternal socioeconomic status and health [23–25]. Most references are based on populations that include fetuses from complicated pregnancies and fetuses with congenital anomalies, which can exacerbate between country and ethnic-specific differences. The consistency between the INTERGROWTH-21^st^ Project standards [1,2] and the WHO Child Growth Standard [3] at birth, and the lack of ethnic-specific differences in fat free measures of newborn size [1,2] also support the concept of a common universal standard. However, the specific centile cut-offs to be used for designating fetuses/newborns as high-risk in any population will need to be determined on an ad hoc basis through a methodology that balances risks, costs and benefits of antenatal and postnatal screening and the resources required for monitoring high-risk subpopulations. Even if the centile cut-offs used to designate abnormal growth vary by country and ethnicity, use of a common standard (i.e., INTERGROWTH) could help standardize fetal and newborn growth assessment and potentially delineate between-country and ethnic-specific differences.

One finding of note was the poor prognostic performance of the small-for-gestational age and large-for-gestational age centiles of both the INTERGROWTH standard and the Canadian reference for identifying infants at high risk for severe neonatal morbidity and neonatal death. Only 7.8% of infants with composite severe neonatal morbidity/mortality were identified by the small-for-gestational age (<10 centile) category of the INTERGROWTH standard, while the Canadian reference identified 14.1% of such infants as small-for-gestational age (<10^th^ centile, Table 4). The sensitivity rates in the large-for-gestational age category were equally poor, with INTERGROWTH criteria >90^th^ centile identifying 21.5% and Canadian criteria identifying 11.8% of composite neonatal morbidity/mortality. These low sensitivity rates are accompanied by low rates of severe neonatal morbidity/mortality in the <3^rd^, <10^th^, >90^th^ and >97^th^ centile categories of both the INTERGROWTH standard and the Canadian reference. The latter implies poor specificity (i.e., a high false positive rate since most infants <3^rd^ centile did not suffer severe neonatal morbidity/death). Growth centiles are perhaps best viewed as one input for use in multivariable models for the screening and identification of high risk infants. Obstetric intervention for abnormal fetal growth is ideally guided by multivariable models that include fetal growth centiles and other risk factors such as uterine and middle cerebral artery blood flow [26–28].

We evaluated the INTERGROWTH newborn standard using a population-based cohort of live births and our findings are likely generalizable to other high income countries with similar birth weight-for-gestational age profiles. It is also likely that our findings and inferences regarding the INTERGROWTH birth weight-for-gestational age standard apply equally to the INTERGROWTH (ultrasound) fetal growth standard [29]. In fact, a case could be made for using the centiles for identifying small- and large-for-gestational age infants (determined through the outcome-based methods proposed above) for fetuses as well since outcome-based determination of biometric centiles for identifying high risk fetuses will be challenging for various reasons including the multiplicity of fetal measurements.

## Conclusion

Adopting the INTERGROWTH-21^st^ Project newborn standard using traditional centile cut-offs for identifying abnormal growth will lead to substantial changes in the identification of small-for-gestational age and large-for-gestational age infants in Canada. Although widespread adoption of the INTERGROWTH standards will standardize fetal and newborn growth assessment, further outcome-based research is required to define the appropriate INTERGROWTH centiles for identifying abnormally growth in fetuses and infants that require increased clinical monitoring and surveillance. Multivariable prognostic models based on INTERGROWTH centiles and other factors indicating fetal and infant well-being should be developed for better assessing fetal and infant health status.

## References

[1] Villar J, Cheikh Ismail L, Victora CG, Ohuma EO, Bertino E, et al International Fetal and Newborn Growth Consortium for the 21st Century (INTERGROWTH-21st). International standards for newborn weight, length, and head circumference by gestational age and sex: the Newborn Cross-Sectional Study of the INTERGROWTH-21st Project. Lancet 2014;384:857–68. 10.1016/S0140-6736(14)60932-6 25209487

[2] Papageorghiou AT, Ohuma EO, Altman DG, Todros T, Cheikh Ismail L, et al International Fetal and Newborn Growth Consortium for the 21st Century (INTERGROWTH-21st). International standards for fetal growth based on serial ultrasound measurements: the Fetal Growth Longitudinal Study of the INTERGROWTH-21st Project. Lancet 2014;384:869–79. 10.1016/S0140-6736(14)61490-2 25209488

[3] WHO Multicentre Growth Reference Study Group. WHO Child Growth Standards: Growth velocity based on weight, length and head circumference: Methods and development. Geneva: World Health Organization, 2009 (242 pages).

[4] Villar J, Papageorghiou AT, Pang R, Ohuma EO, Cheikh Ismail L, et al International Fetal and Newborn Growth Consortium for the 21st Century (INTERGROWTH-21st). The likeness of fetal growth and newborn size across non-isolated populations in the INTERGROWTH-21st Project: the Fetal Growth Longitudinal Study and Newborn Cross- Sectional Study. Lancet Diabetes Endocrinol 2014;2:781–92. 10.1016/S2213-8587(14)70121-4 25009082

[5] Kramer MS, Platt RW, Wen SW, Joseph KS, Allen A, Abrahamowicz M, et al A new and improved population-based reference for birth weight for gestational age. Pediatrics 2001;108: e35 11483845 10.1542/peds.108.2.e35

[6] Wen SW, Liu S, Marcoux S, Fowler D. Uses and limitations of routine hospital admission/ separation records for perinatal surveillance. Chronic Dis Can 1997;18:113–9. 9375258

[7] Joseph KS, Fahey J. Validation of perinatal data in the Canadian Institute for Health Information’s Discharge Abstract Database. Chron Dis Can 2009;29:96–100. 19527567

[8] Janes H, Pepe MS, Gu W. Assessing the value of risk predictions by using risk stratification tables. Ann Intern Med 2008;149:751–60. 19017593 10.7326/0003-4819-149-10-200811180-00009 PMC3091826

[9] Joseph KS. The natural history of pregnancy: diseases of early and late gestation. BJOG 2011;118:1617–29. 10.1111/j.1471-0528.2011.03128.x 21895957

[10] Seeds JW, Peng T. Impaired growth and risk of fetal death: is the tenth percentile the appropriate standard? Am J Obstet Gynecol 1998;178:658–69. 9579427 10.1016/s0002-9378(98)70475-2

[11] Tyson JE, Kennedy K, Broyles S, Rosenfeld CR. The small for gestational age infant: accelerated or delayed pulmonary maturation? Increased or decreased survival? Pediatrics 1995;95:534–38. 7700754

[12] Bernstein IM, Horbar JD, Badger GJ, Ohlsson A, Golan A. Morbidity and mortality among very-low-birth-weight neonates with intrauterine growth restriction. Am J Obstet Gynecol 2000;182:198–206. 10.1016/s0002-9378(00)70513-8 10649179

[13] Yoon JJ, Kohl S, Harper RG. The relationship between maternal hypertensive disease of pregnancy and the incidence of idiopathic RDS. Pediatrics 1980;65:735–9. 7367080

[14] Piper JM, Xenakis EM, McFarland M, Elliott BD, Berkus MD, Langer O. Do growth- retarded premature infants have different rates of perinatal morbidity and mortality than appropriately grown premature infants? Obstet Gynecol 1996;87:169–74. 10.1016/0029-7844(95)00400-9 8559517

[15] Evans N, Hutchinson J, Simpson JM, Donoghue D, Darlow B, Henderson-Smart D. Prenatal predictors of mortality in very preterm infants cared for in the Australian and New Zealand Neonatal Network. Arch Dis Child Fetal Neonatal Ed. 2007;92:F34–40. 10.1136/adc.2006.094169 16877475 PMC2675296

[16] Francis JH, Permezel M, Davey MA. Perinatal mortality by birthweight centile. Aust N Z J Obstet Gynaecol. 2014;54:354–9. 10.1111/ajo.12205 24731210

[17] Vasak B, Koenen SV, Koster MP, Hukkelhoven CW, Franx A, Hanson MA, et al Human fetal growth is constrained below optimal for perinatal survival. Ultrasound Obstet Gynecol 2015;45:162–7. 10.1002/uog.14644 25092251

[18] Joseph KS, Fahey J, Platt RW, Liston RM, Lee SK, Sauve R, et al An alternate approach to the creation of fetal growth standards: Do singletons and twins need separate standards? Am J Epidemiol 2009;169:616–24. 10.1093/aje/kwn374 19126584 PMC2640160

[19] Steer PJ. Possible differences in fetal size by racial origin. Lancet Diabetes Endocrinol 2014; 2:766–7. 10.1016/S2213-8587(14)70157-3 25009083

[20] Gardosi J. Fetal growth and ethnic variation. Lancet Diabetes Endocrinol 2014;2:773–4. 10.1016/S2213-8587(14)70188-3 25258203

[21] Albert PS, Grantz KL. Fetal growth and ethnic variation. Lancet Diabetes Endocrinol 2014;2:773. 10.1016/S2213-8587(14)70186-X PMC10428134 25258202

[22] Kramer MS. Does one size fit all? Should India Adopt the New INTERGROWTH-21^st^ “Prescriptive” Standard for Fetal Growth? Bulletin of the Nutrition Foundation of India. 2015;36(3):1–5.

[23] Unterscheider J, Daly S, Geary MP, Kennelly MM, McAuliffe FM, O'Donoghue K, et al Optimizing the definition of intrauterine growth restriction: the multicenter prospective PORTO Study. Am J Obstet Gynecol 2013;208:290.e1–6. 23531326 10.1016/j.ajog.2013.02.007

[24] Copel JA, Bahtiyar MO. A practical approach to fetal growth restriction. Obstet Gynecol 2014;123:1057–69. 10.1097/AOG.0000000000000232 24785859

[25] Seravalli V, Baschat AA. A uniform management approach to optimize outcome in fetal growth restriction. Obstet Gynecol Clin North Am 2015;42:275–88. 10.1016/j.ogc.2015.01.005 26002166

[26] Kramer MS. Socioeconomic determinants of intrauterine growth retardation. Eur J Clin Nutr 1998;52:S29–32; discussion S32-3. 9511017

[27] Mortensen LH, Diderichsen F, Davey Smith G, Nybo Andersen AM. Time is on whose side? Time trends in the association between maternal social disadvantage and offspring fetal growth. A study of 1 409 339 births in Denmark, 1981–2004. J Epidemiol Community Health 2009;63:281–5. 10.1136/jech.2008.076364 19147631

[28] Black RE, Victora CG, Walker SP, Bhutta ZA, Christian P, de Onis M, et al Maternal and Child Nutrition Study Group. Maternal and child undernutrition and overweight in low- income and middle-income countries. Lancet 2013;382:427–51. 10.1016/S0140-6736(13)60937-X 23746772

[29] Perinatal Services BC Standards for Obstetrical Ultrasound Assessments. Perinatal Services BC. Provincial Health Services Authority. Vancouver, British Columbia, Canada. 2015 Available at http://www.perinatalservicesbc.ca/Documents/Guidelines-Standards/Standards/Ultrasound/PSBCUltrasoundAssessmentStandards.pdf (accessed February 4, 2016).

